# 74 The Association Between Burn Injury and Peer Relations: A preschool-libre1-5 Study

**DOI:** 10.1093/jbcr/irac012.077

**Published:** 2022-03-23

**Authors:** Khushbu F Patel, Pengsheng Ni, Silvanys L Rodríguez-Mercedes, Kate E Surette, Camerin A Rencken, Renata Fabia, Carrie Tully, Tina L Palmieri, Kathleen S Romanowski, Petra Warner, Frederick J J Stoddard, Jeffrey C Schneider, Lewis E Kazis, Colleen M Ryan

**Affiliations:** Shriners Hospitals for Children - Boston/MGH, Boston, Massachusetts; Boston University School of Public Health, Boston, Massachusetts; Shriners Hospitals for Children - Boston, Boston, Massachusetts; Shriner's Hospitals for Children - Boston, Boston, Massachusetts; Shriners Hospitals for Children - Boston, Seattle, Washington; Nationwide Children's Hospital, Columbus, Ohio; Children's National Hospital, Washington, District of Columbia; UC Davis and Shriners Hospitals for Children Northern California, Sacramento, California; University of California, Davis and Shriners Hospitals for California Northern California, Sacramento, California; Shriners Children's Ohio, Dayton, Ohio; Harvard Medical School, Boston, Massachusetts; , Massachusetts; Boston University School of Public Health, Spaulding Rehabilitation Hospital, Harvard Medical School, Boston, Massachusetts; Harvard Medical School, Boston, Massachusetts

## Abstract

**Introduction:**

Children ages one to five years old are naturally curious and build their personality and social skills through interactions with others. Positive peer relations are especially important after a burn injury as bullying and peer rejection can delay development of social skills. This study assessed the association between burn injuries and burn survivors’ ability to connect with and maintain peer relations in this age group using the Preschool-LIBRE _1-5_ (Life Impact Burn Recovery Evaluation).

**Methods:**

The Preschool-LIBRE_1-5_ was field-tested with 426 parents of burn survivors. Each item was scored on a 5-point Likert scale ranging from 0 (never) to 4 (always). Data was recoded for selected items such that higher scores denote better functioning. Classic test theory methods were used to assess the peer relation items from a social functioning domain. Individual items and mean scores in the domain were examined. Multiple linear regression analyses (controlling for gender, race and ethnicity, pain severity, burn injury to critical area, burn size, and age at survey completion) measured the association between demographic and clinical characteristics and calculated a peer relation score based on multiple imputation samples.

**Results:**

The mean age was 3.06 + 1.41 years, mean time since injury of 1.16 + 1.34 years, mean total body surface area (TBSA%) of 4.21 + 7.92, and 55.16% male and 74.18% white. Items from peer relations item pool (n=15) were identified as a unidimensional scale (α=0.92, item-total correlations for all 15 items >0.4, ratio of the 1st and 2nd eigenvalues (8.729/1.287=6.78) = > 4). The mean peer relation score was 2.86 + 0.76. The two items with the lowest and highest score were “My child would ask for things nicely when playing with other children” (x̄ = 2.09) and “My child liked to play near and be with family members and friends (x̄ = 3.59) respectively. Results indicated that age was a significant predictor, such that older age at survey completion was significantly associated with higher peer relation score (β = 0.16, *p* < 0.0001). With each year of age increase, peer relationship score increased by 0.16 + 0.21 points.

**Conclusions:**

Preschool-aged burn survivors, as reported by parents, often had the ability to connect with peers through imitation and participating in play activities, and maintained peer relationships well. These findings emphasize the importance of promoting early interventions that build social skills, allowing for positive interactions with peers and improving social functioning in the long-term.

